# The Long-Pulse Potassium-Titanyl-Phosphate Laser: Promising Treatment for Resistant Port-Wine Stains

**DOI:** 10.7759/cureus.53994

**Published:** 2024-02-11

**Authors:** Rawan Almutairi, Saima Usmani, Sara Hussein, Wael Aldaraji

**Affiliations:** 1 Dermatology, Farwaniya Hospital, Ministry of Health, Farwaniya, KWT; 2 Dermatology, My Skin Clinic, Kuwait City, KWT; 3 Dermatology, Laser and Skin Clinic, Baghdad, IRQ; 4 Dermatology, The Ghanem Clinic, London, GBR

**Keywords:** laser, long pulse ktp, pdl-resistance, pdl, pws

## Abstract

Port-wine stains (PWSs), or port-wine birthmarks, are congenital vascular malformations that manifest as erythematous to pink patches at birth. At present, lasers are the preferred method for treating PWSs, with pulsed dye laser (PDL) being regarded as the gold standard because of its superior efficacy compared to alternative procedures. Despite the progress made in laser therapy, a subset of patients continue to experience PWSs that cannot be resolved effectively even with PDL. A new long-pulse potassium-titanyl-phosphate (KTP) laser with a trail of sub-pulses (Derma V, Lutronic, Seoul, South Korea) is a promising treatment for PWSs resistant to PDL therapies. This is a case of a female patient with PDL-resistance PWSs that was treated successfully with a long-pulse KTP laser. Long-pulse KTP appears to be not just more effective in treating PDL-resistant PWSs but also less costly as less number of sessions are needed, with no significant side effects reported such as purpura.

## Introduction

Port-wine stains (PWSs), or port-wine birthmarks, are congenital vascular malformations that manifest as erythematous to pink patches at birth. They affect an equal proportion of males and females, ranging from 0.3% to 0.5% of the population [1]. The aetiology and cause of PWSs remain poorly understood, and the most probable explanation for the occurrence of PWSs is the insufficiency or absence of surrounding neurons that control blood flow via ectatic postcapillary venules. Consequently, the blood vessels remain perpetually dilated and incapable of constricting normally [2]. The haemoglobin pigment gives them a deep reddish purple to violaceous colour that resembles Port-Wine, hence their name [3]. The psychosocial development and well-being of patients may be adversely affected by PWSs, as 70-80% of the lesions manifest on the face and neck. This is primarily due to the patients' awareness of their atypical cosmetic appearance [4]. Skin grafting, electrotherapy, cryosurgery, derma-abrasion, and cosmetic tattooing are all types of treatment modalities for PWSs; nevertheless, none of these alternatives appear to be effective and are incapable of producing consistent, satisfactory results, or even complete remedies. At present, lasers are the preferred method for treating PWSs, with pulsed dye laser (PDL) being regarded as the gold standard because of its superior efficacy compared to alternative procedures [5].

Despite the progress made in laser therapy, a subset of patients continue to experience PWSs that cannot be resolved effectively even with PDL. When conditions are resistant to conventional laser therapy, an alternative treatment method may be necessary. A new long-pulse potassium-titanyl-phosphate (KTP) laser with a trail of sub-pulses (Derma V, Lutronic, Seoul, South Korea) is a promising treatment for PWSs resistant to PDL therapies. Herein, we present a case of a female patient with PDL-resistance PWSs who was treated successfully with a long-pulse KTP laser.

## Case presentation

**Figure 1:**
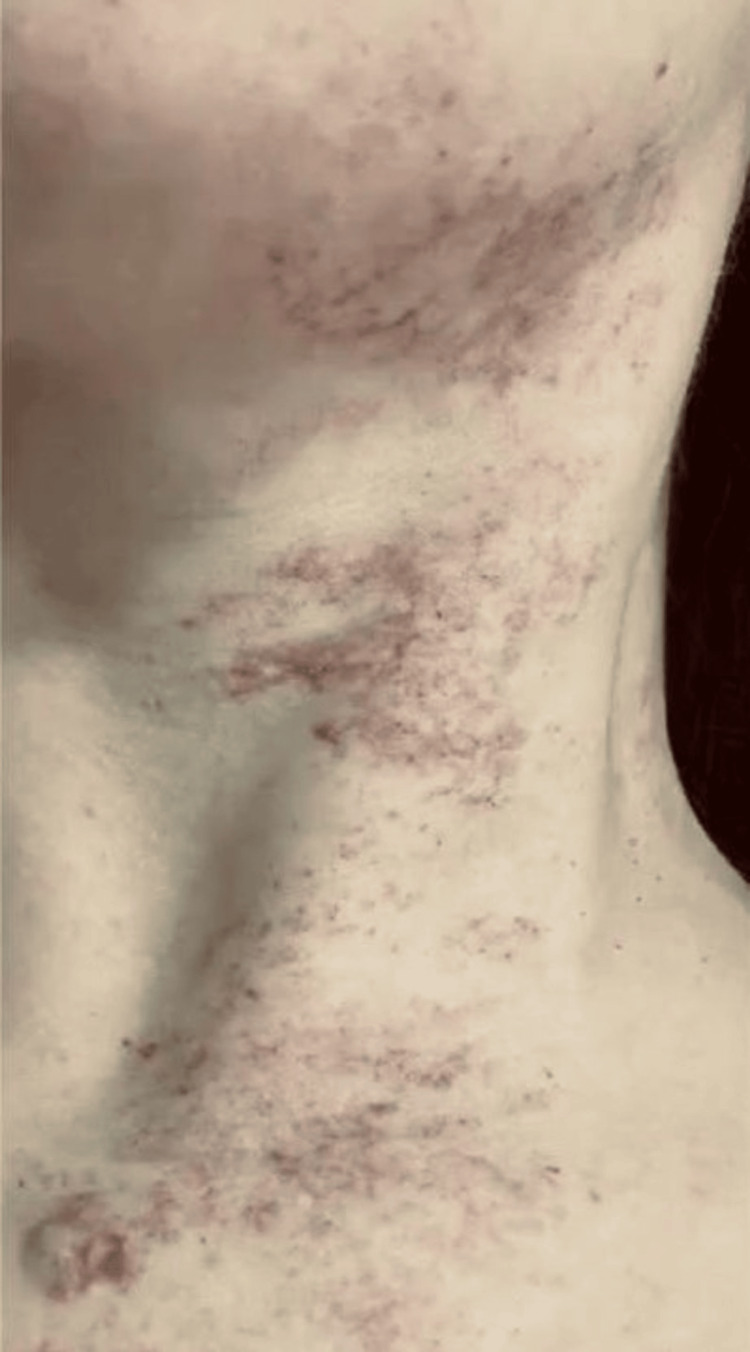
Port-wine stains on the anterior region of the neck Flat, red to purple lesions on the anterior neck region, representing port-wine stains.

**Figure 2:**
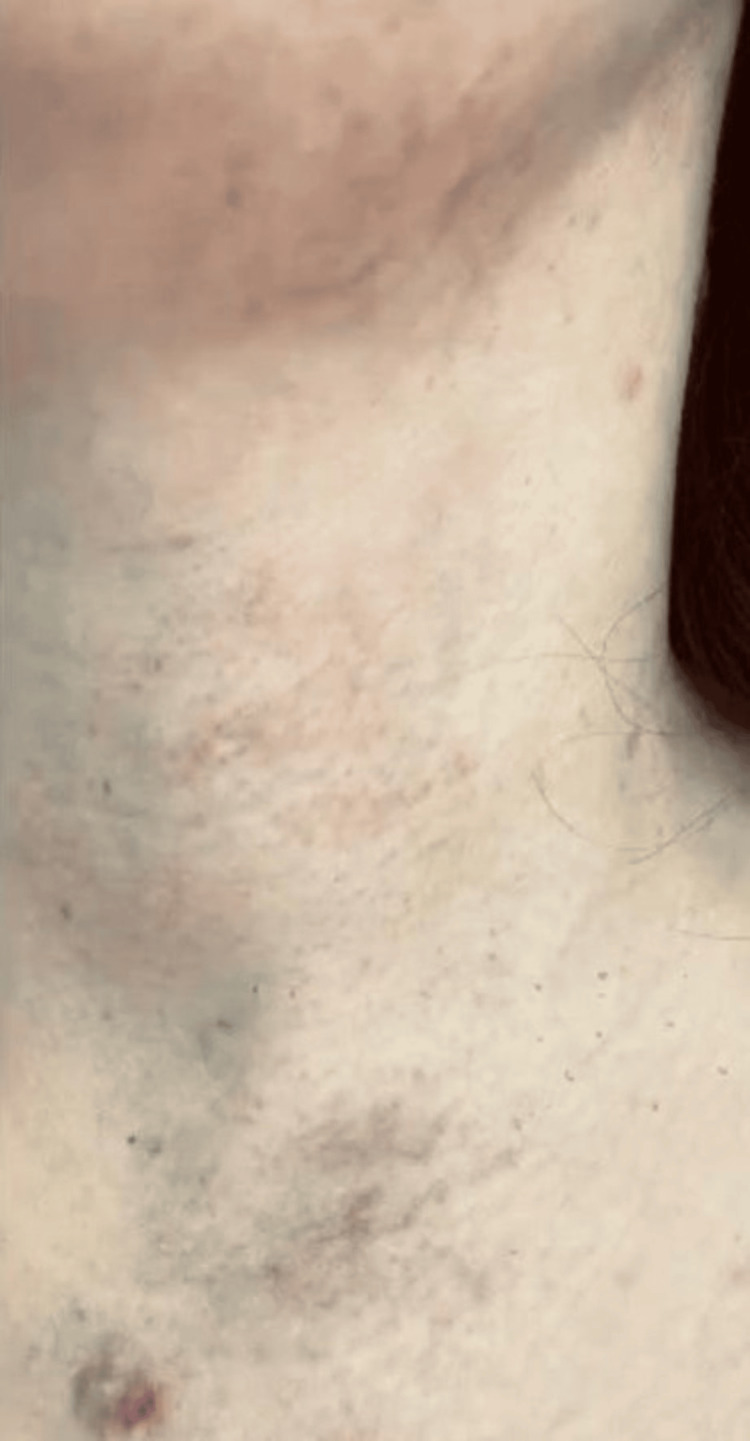
Port-wine stains on the anterior region of the neck The same lesions after three sessions of long-pulse potassium titanyl phosphate (KTP) laser non-purpuric dose.

A 30-year-old female patient, skin type 3, presented to the dermatology clinic for an evaluation of PWSs on the anterior neck region, since the age of two years (Figure 1). She had seven laser sessions done in other clinics with unknown settings. The laser used was purpuric doses of PDL. The patient reported some improvement but was unsatisfactory and on a couple of occasions, she had post-inflammatory hyperpigmentation which lasted two to three months. We treated her with only three sessions of derma V non-purpuric dose over three to four months using this setting (12mm, 10ms (Sub Micro), 5-7 J/cm^2^, ICD 15/15/10 (non-purpuric)). The patient showed more than 50% improvement in her skin lesions, with no complications after the sessions (Figure 2), and was satisfied with the final result. Two blinded dermatologists, independent of the primary treating physician, compared the figures and categorised the improvement into four ranges: <25%, 25-50%, 50-75%, and >75%. Both agreed on 50% to 75% improvements.

## Discussion

Several factors affect the treatment response of PWSs. Patients with lighter skin types had a better treatment response. PWSs located in the face and neck exhibited a more favourable response than those located in the extremities. Furthermore, PWSs on the lateral face reported a more favourable response than those on the central face. Proximal extremity lesions also responded better than distal extremity lesions did. The increased susceptibility to blistering and scarring of the neck and eyelids should be considered when determining the appropriate laser parameters. Pink, red, and reticular lesions responded better than purple and geographically shaped lesions. Not unexpectedly, PWSs with overgrowth will show a lesser response than those that are flat, smooth, and not associated with contour changes [3,6,7]. Although PWSs on the face and neck responded better than those on the extremities, restoration of our patient’s PWSs on the neck failed multiple sessions of PDL.

KTP lasers are quasi-continuous lasers that emit at a wavelength of 532 nm and are well absorbed by haemoglobin. This is achieved using neodymium: yttrium-aluminium-garnet (Nd: YAG) crystal frequency doubled with that of a KTP crystal. Spot diameters up to 5 mm, fluences up to 240 J/cm^2^, and pulse durations ranging from 1 to 150 ms are the characteristics of such a laser. It can be utilized to treat small vessels, including facial telangiectasias, because it generates energy pulses with small spot sizes. This laser can have scanning devices attached to it so that non-overlapping pulses are distributed over wider areas [8].

KTP lasers have been widely used in various medical applications, including dental procedures, laryngeal surgery, skin rejuvenation, and prostate surgery. Its effectiveness in killing bacteria such as *Enterococcus faecalis* has been demonstrated, making it a potential tool for root canal treatment [9]. Additionally, the KTP laser has shown promise in the treatment of laryngeal diseases such as papilloma and dysplasia, owing to its photoangiolytic properties. Surgeons found that the KTP laser for laryngeal lesions and vocal fold vascular lesions is still relatively innovative and is equivalent to or superior to PDL, and none have reported inferiority [10]. In prostate surgery, a KTP laser has been employed for photoselective vaporization, offering a promising option with evolving technologies [11]. Furthermore, it has been used in skin rejuvenation, demonstrating improvements in redness, pigmentation, skin tone, and texture [12].

The advantage of the KTP is that target vessels are not ruptured, leading to much less purpura compared to PDL. This was due to the pulse duration. Longer pulse lengths allow laser energy to be delivered to vessels for extended periods of time, which reduces vessel rupture and the hyperpigmentation that accompanies it while producing mild, homogeneous heating or coagulation over the entire vessel. With a pulse duration reaching 150 ms, the KTP laser was the first laser able to reliably remove linear vessels without purpura, producing vessel blanching at the same time of treatment. Yet, PDLs were unable to create continuous pulses longer than 1.5 ms without the use of subpulses [8,13]. Interestingly, PDLs emitting 585 nm and short pulse durations of 0.45 ms were initially developed to treat PWSs, however, PDL always left a week or more of significant purpura [14].

Since KTP lasers are considered the standard treatment for different skin lesions due to their unique strong laser energy absorption by haemoglobin and minimal effect on normal skin tissues. However, the high absorption of the laser by epidermal melanin could lead to unwanted permanent thermal complications such as hypertrophic scarring and dyspigmentation. This was eliminated by the introduction of cryogen spray cooling. Cryogen spray cooling has been extensively studied and applied in dermatologic laser surgery to protect the epidermis during various treatments. Its selective cooling effect on the superficial layers of the skin makes it a valuable technology for minimizing thermal injury during laser therapy [12,15].

## Conclusions

Long-pulse KTP appears to be not just more effective in treating PDL-resistant PWSs but also less costly as less number of sessions are needed, with no significant side effects reported. In order for PDL to be most effective, it should produce purpura and then the associated post-inflammatory hyperpigmentation. Yet, this wasn’t detected in our case with the use of long-pulse KTP, which is considered as a new advantage of KTP laser.
